# An Analysis of Sponsors/Collaborators of 69,160 Drug Trials Registered with ClinicalTrials.gov

**DOI:** 10.1371/journal.pone.0149416

**Published:** 2016-02-17

**Authors:** Shruthi Muralidharan Keezhupalat, Ankeet Naik, Saurabh Gupta, Raghunathan Srivatsan, Gayatri Saberwal

**Affiliations:** 1 Institute of Bioinformatics and Applied Biotechnology, Bengaluru, Karnataka, India; 2 GANIT Labs, Institute of Bioinformatics and Applied Biotechnology, Bengaluru, Karnataka, India; Renal Division, Peking University First Hospital, CHINA

## Abstract

**Background:**

Clinical trials have been criticized on various counts. Any attempt to improve how trials are conducted or reported requires—amongst other things—an understanding of the number, the nature and the location of those that sponsor them or collaborate on them. Here we sought to identify the nature and location of each sponsor/collaborator.

**Methods and Findings:**

We examined the 'sponsor/collaborator' field for the 69,160 drug trials that were registered with ClinicalTrials.gov over a 9-year period (2005–2014). Of the 12,823 unique sponsors, 56% had sponsored only one and 27% had sponsored 2–5 trials each. Just 18% were involved with six or more trials each, and we have (arbitrarily) labeled these organizations as 'more experienced' in sponsoring/collaborating on trials. These 18% (2,266 sponsors/collaborators) were analyzed further: (a) 951 were corporate organizations and (b) 1,145 were non-corporates (including 31 individuals) with (c) 170 unclassified. Further, we identified the location of each organization in (a) and (b).

**Conclusions:**

Clinical trials are an important part of a nation's research endeavors, and ultimately contribute to the health of its people. Thus, understanding the clinical trial landscape—including the number and nature of sponsors, and how active they are—is important for every country. We believe that policy makers in particular should be interested in this study to understand the current situation, and to use the numbers as a baseline for the evolving landscape, to assess the impact of their strategies in future.

## Introduction

Clinical trials have been criticized on various counts. They may fail to follow the guidelines of the International Committee of Medical Journal Editors (ICMJE) [1,2]. Trials are often unable to recruit the planned number of participants per site, leading to an over-dependence on a few sites [3,4]. Even so the trials may have too few participants to yield useful information [5]. Further, it has been estimated that in a large percentage of cases it is the wrong test dose that leads to a failed trial [6]. Often, those conducting a trial have insufficient input from patient organizations [7], may not keep patients informed of the results [8] and may not take enough steps to enroll minorities [9]. Additionally, they may be unaware that subjects are not taking the test drug according to the protocol, or may be enrolled in multiple trials at the same time [10]. Trials may take too long for the participants to benefit [11]. Early results from trials may be leaked [12]. In terms of reporting the results, there may be a publication bias that exaggerates the benefits of a drug candidate or minimizes its risks [13, 14], peer-review of randomized controlled trials may not be up to the mark [15] and journals may not meet the required standards while reporting the results of trials [16].

Such criticism has often been directed at trials conducted in traditional locations such as the United States (US) or Western Europe. However trials are increasingly located in non-traditional nations [17, 18]. Problems reported from some of these locations include the lack of a control group, of informed consent and of a system to report adverse events [19], over- or under-monitoring of a trial [18], reduced safety requirements by the Food and Drug Administration of the US (USFDA) for trials conducted abroad [20], the inexperience of local investigators [21] and insufficient local leadership in conducting trials [22], leading to doubts about their quality [21].

Any attempt to improve how trials are conducted or reported requires—amongst other things—an understanding of the number, the nature and the location of those that sponsor them or collaborate on them. In this study we sought to understand this landscape.

## Methods

ClinicalTrials.gov (accessed at http://clinicaltrials.gov and hereafter referred to as CT.gov) is the largest registry of clinical trials [23]. In the information downloaded from CT.gov, we were interested in the 'sponsor/collaborator' field. If a record had multiple names in this field, we treated them at par. For convenience we referred to each organization as a sponsor since there was no way to more precisely identify an organization's role(s).

We accessed CT.gov on 4 November 2015 and did an Advanced Search, with the following filters: (i) 'Study type': Interventional studies; (ii) 'Phase': 0–4; and (iii) 'Record first received': 1/1/2005 to 01/31/2014. This yielded 85,528 records that we downloaded with the options '22 available fields' and as 'Tab-separated values'. From these, we selected the drug-related records, that is those that had as 'intervention' either 'drug' or 'biological'. These came to 69,160 records. This file was processed to yield (a) the list of 107,911 sponsors, including redundancies and (b) the list of 12,823 unique sponsors and their frequencies (S1 Table). After identifying this very large number of sponsors/collaborators, we focused on those that had been involved in six or more trials. We categorized each of such organizations as 'corporate' or 'non-corporate'. We also identified the location of each (except the 31 individuals amongst the non-corporates). In some cases we could not conclusively identify the nature of the organization or its location and these were excluded from further analysis. S1 Text provides further details of the methodology.

## Results and Discussion

**Fig 1:**
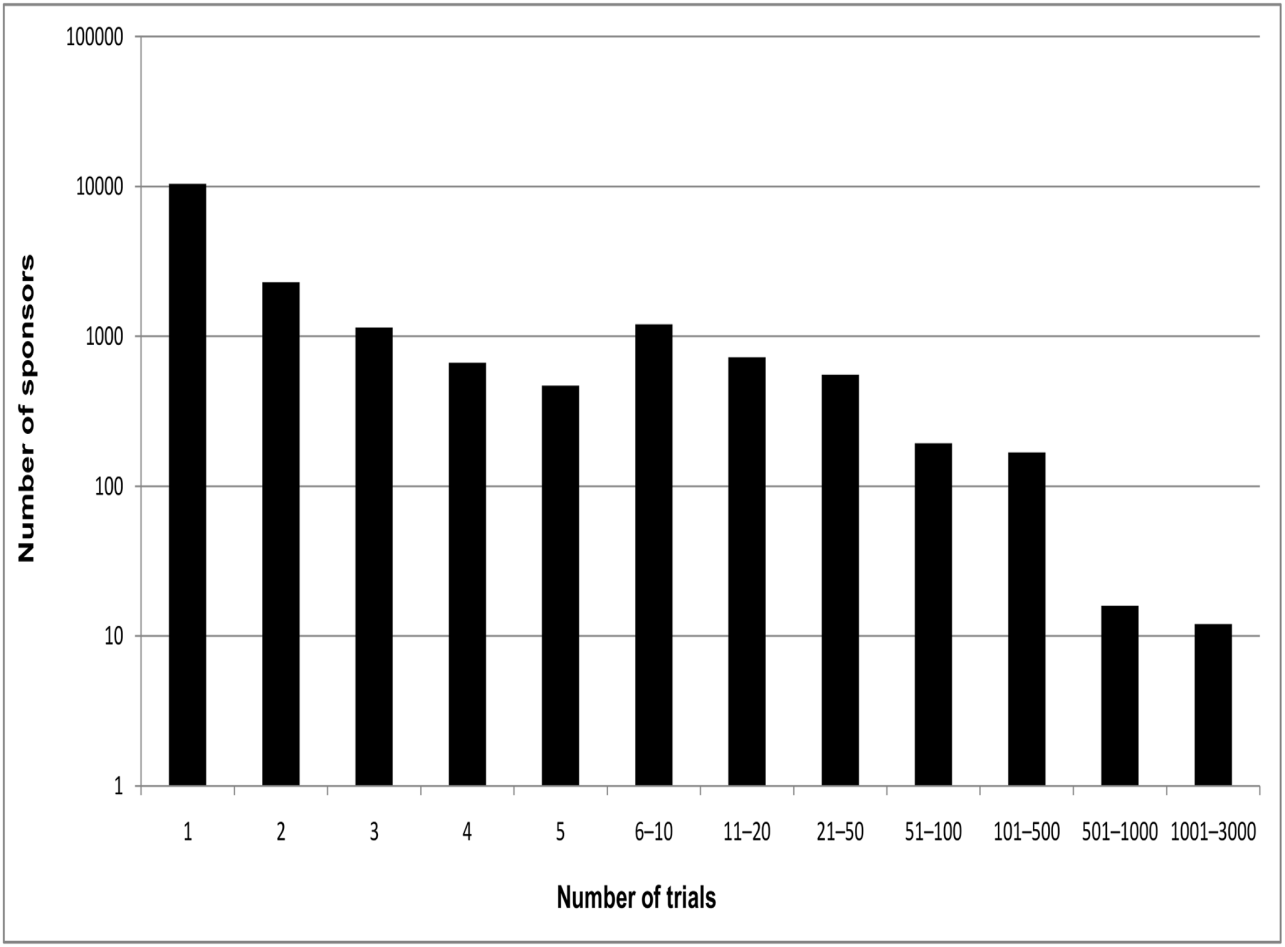
The distribution of sponsors over different numbers of trials. The sponsor with the largest number of trials (the National Cancer Institute, with 3,498) is not shown.

Fig 1 provides a summary of the frequency of occurrence of the sponsors. Of the 12,823 unique sponsors, 7,119 (56%) were involved in a single trial each. A further 3,438 (27%) were associated with 2–5 trials. This left just 2,266 (18%) sponsors that were involved in six or more trials. For convenience we refer to the 2,266 organizations as those with 'more experience', and the rest as those with 'limited experience' as sponsors. Many studies were co-sponsored by multiple organizations, and a significant percentage must have involved a partnership between sponsors with more experience and those with limited experience.

10,557 organizations sponsored 1–5 trials each, totaling 17,081 trials. We did not analyze this group any further. We sought to classify each of the remaining 2,266 (listed in S2 Table and sorted in S3 Table) as corporate or non-corporate, with the latter including non-profits, government organizations, individuals or 'others' (with the criteria for classification provided in S2 Text). Trials were sponsored by 951 corporates (45,532 occurrences overall) and 1,145 non-corporates (41,812). For 170 entities (3,486) the nature of the organization or its location could not be conclusively determined and they were labeled 'unclassified'. In all, the 2,266 organizations occurred 90,830 times which is 84% of the 107,911 incidences of sponsorship. Thus, the fraction of sponsors with more experience was low (18%), but the fraction of trials in which these experienced sponsors were involved was high (84%).

Corporates occurred 45,532 times and non-corporates 41,812 times, which were, 50% and 46% respectively of the 90,830 total number of occurrences. Since there have been disagreements over whether the private or public sector brings drugs to market [24], these results would be useful to any attempt to quantify the relative contributions of the two sectors. However it is true that without knowing precisely what each organization did, it is impossible to quantify the costs that the two sets of organizations incurred, for example. Separately, a non-profit may have close ties with industry, and in fact may be the non-profit wing of a company. We have not documented the extent of such relationships in this paper.

**Fig 2:**
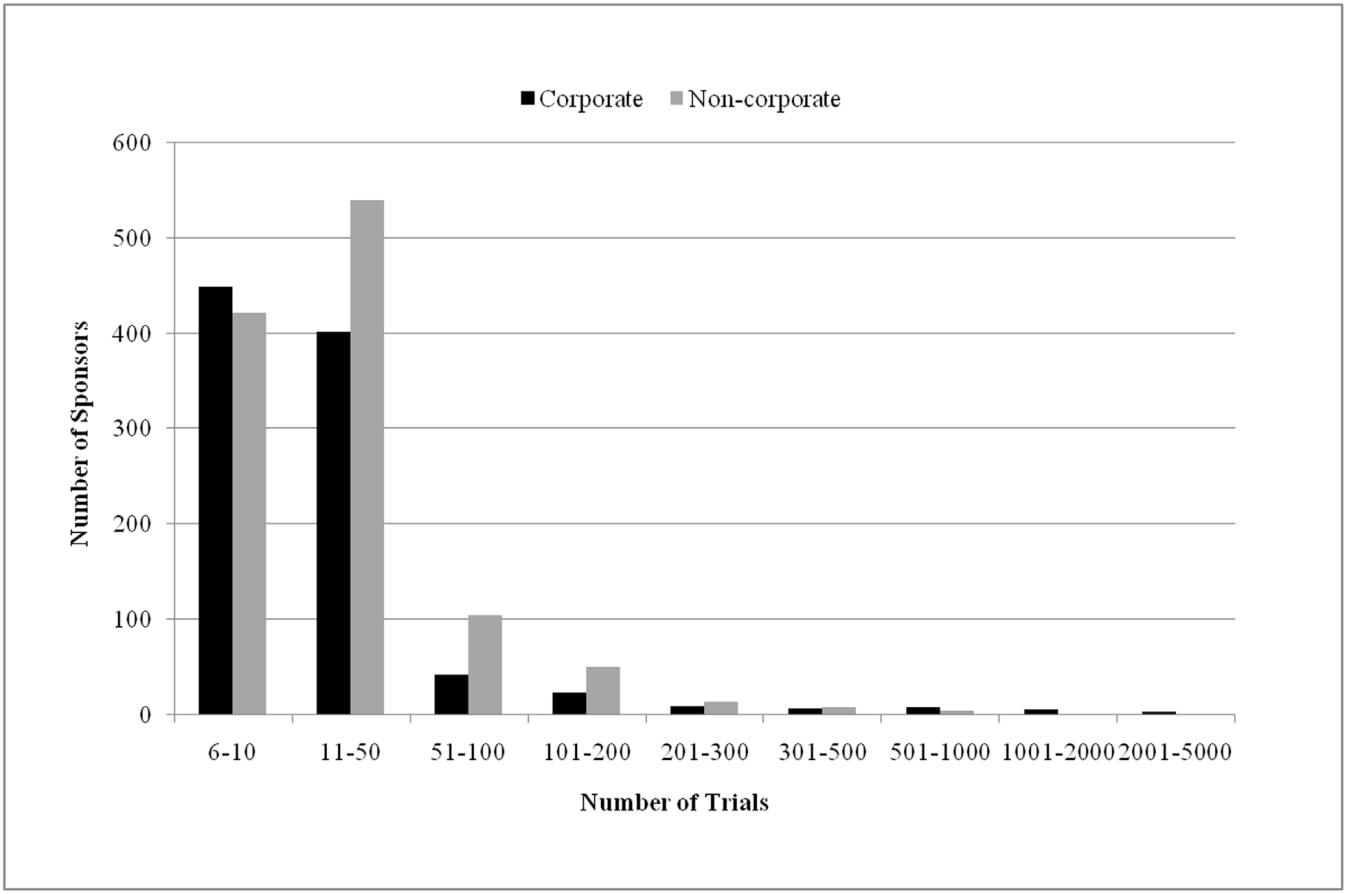
The numbers of corporates and non-corporates that sponsored a given number of trials.

We continued our analysis of the organizations that had sponsored six or more trials each and looked at how many trials were sponsored by the 951 corporates and 1,145 non-corporates (Fig 2). 90% of corporate-sponsored and 84% of non-corporate sponsored studies were by organizations involved in no more than 50 trials each. Only 57 corporates and 78 non-corporates sponsored more than 100 trials each. Further, just a few sponsored more than 1,000 each. The nine corporates were Hoffmann-La Roche (1,058), Eli Lilly and Company (1,269), Novartis Pharmaceuticals (1,439), AstraZeneca (1,619), Sanofi (1,622), Merck Sharp & Dohme Corp. (1,811), Pfizer (2,313), Novartis (2,487) and GlaxoSmithKline (2,930). The non-corporate that did so was the National Cancer Institute (3,498). Thus, the vast majority of sponsors—corporate or non-corporate—were involved in relatively few trials.

Finally, we identified the countries in which the corporate and non-corporate sponsors were based (methodology in S2 Text). In all, the corporates were based in 36 countries and the non-corporates in 63. We list here the top 12 locations for each category (Table 1, with the complete lists in S4 Table). Amongst the corporates, the US was the base for 56% of the sponsors. Also, it was home to an order of magnitude more organizations than the second ranked country, Germany (6%). South Korea, Japan and China are the only Asian countries on this list, with the rest being developed, Western countries. These 12 countries were home to 87% of the corporate sponsors. Among the non-corporates, the US was the base for 33% of the organizations and hosted almost five fold more sponsors than the second-placed country, China (7%). China and Brazil are the two developing countries that made it to this list. The top 12 locations hosted 74% of the sponsors. The dominance of the US in both lists parallels a report that it dominates the 'trials' locations' table, with almost 9-fold more sites than the second-ranked Germany [25]. However countries that have not traditionally been involved in clinical trials made their presence felt.

**Table 1:** The 12 countries that were home to the most sponsors in the corporate and non-corporate categories.

No.	Corporates				Non-corporates			
	Country	Number of sponsors	Percentage	Cumulative percentage	Country	Number of sponsors	Percentage	Cumulative percentage
1	US	529	55.6	55.6	US	380	33.2	33.2
2	Germany	53	5.6	61.2	China	79	6.9	40.1
3	South Korea	34	3.6	64.8	UK	59	5.2	45.2
4	Canada	31	3.3	68	Canada	57	5.0	50.2
5	Japan	31	3.3	71.3	France	57	5.0	55.2
6	UK	31	3.3	74.6	Germany	51	4.5	59.7
7	France	29	3.0	77.6	Italy	47	4.1	63.8
8	Switzerland	24	2.5	80.1	Spain	35	3.1	66.8
9	China	20	2.1	82.2	Denmark	25	2.2	69.0
10	Australia	14	1.5	83.7	Brazil	20	1.7	70.7
11	Israel	14	1.5	85.2	Belgium	19	1.7	72.4
12	Ireland	13	1.4	86.5	Netherlands	19	1.7	74.1
	**Total**	**823**	**87**		**Total**	**848**	**74**	
	**Overall total**	**951**			**Overall total**	**1145**		

## Conclusion

Mainly, this paper provides two categories of information. First, it provides the list of all organizations that were sponsors/collaborators (sponsors) of drug-related interventional trials registered with CT.gov over a recent 9-year period, and the frequency of occurrence of each. Second, it provides the global rankings of countries that hosted (a) the corporate and (b) the non-corporate sponsors that occurred six or more times. We believe that policy makers in particular should be interested in this study to understand the current situation, and to use the numbers as a baseline for the evolving landscape, to assess the impact of their strategies in future.

## Supporting Information

S1 Table The unique sponsors. The 12,823 unique sponsors and the number of trials sponsored by each.
(XLS)

S2 Table Sponsors of six or more trials. The 2,266 organizations that sponsored six or more trials and the number of trials sponsored by each.
(XLS)

S3 Table Categorization of the sponsors of six or more trials. The 2,266 organizations sorted into (a) corporates (951 entities), (b) non-corporates (1,145) and (c) unclassified entities (170).
(XLS)

S4 Table The base country of sponsors. For the (a) corporates and (b) non-corporates, the number of sponsors based in a particular country is listed. The percentage hosted by a given country and the cumulative percentage is also provided for each category.
(XLS)

S1 Text Expanded methods. (A) A note on the records downloaded, (B) Determining the number of sponsors, (C) Classifying each organization, and (D) Determining each organization's location.
(DOC)
