# Supplementary material for: An Analysis of Sponsors/Collaborators of 69,160 Drug Trials Registered with ClinicalTrials.gov
Source: PLoS One. 2016 Feb 17;11(2):e0149416. doi: 10.1371/journal.pone.0149416 (PMC4757412; doi:10.1371/journal.pone.0149416)
Supplement: S1 Text — (A) A note on the records downloaded, (B) Determining the number of sponsors, (C) Classifying each organization, and (D) Determining each organization's location. (DOC) [file pone.0149416.s005.doc]

**S1 Text. Expanded** **methods**. (A) A note on the records downloaded; (B) Determining the number of sponsors; (C) Classifying each organization and (D) Determining each organization's location.

**(A) A note on the records** **downloaded**

We first downloaded 94,018 records on 4 February 2014. Subsequently, in response to a Reviewer's recommendation, we downloaded a narrower set of 85,528 records on 4 November 2015. Thus, most of the work related to classifying organizations and identifying their locations was done in 2014 even though the 'download date' is in November 2015.

**(B) Determining the number of sponsors**

The list of 85,528 trials (with one or more sponsors each) downloaded from CT.gov was processed further by two methods.

*Method 1:*

The list of 85,528 trials downloaded from CT.gov was processed further as follows:

1. 1. To shortlist drug-related trials, those with 'Drug:' and/or 'Biological:' interventions were filtered out from the 85,528 records. First, records with “Drug:' interventions were retrieved. Then, from the remaining data, the records with 'Biological:' intervention were retrieved. These steps was done using the grep command.
   1. grep "Drug:\ " 85528-Original-data.csv > Drug.csv [This contained 63,290 records]
   2. grep "Biological:\ " Without-Drug-.csv > Biological.csv [This contained 5,870 records]
2. 2. The outputs from the two steps above, that is Drug.csv and Biological.csv, were merged to form the file Medicine-trials.csv. This was done using the following command:

cat Drug.csv Biological.csv > Medicine-trials.csv [This contained 69,160 records]

1. 3. For 'incidences of sponsorship', the following command was executed:

cat Medicine-trials.csv| cut -f4 | tr '|' '\n' > Sponsors-new-line.csv [This contained 1,07,911 records]

1. 4. The next step was to count the unique sponsors and their frequencies of conducting trials. For doing this, the output from step 3 was used, that is file 'Sponsors-new-line.csv'. This was processed with the following command:

cat Sponsors-fresh.csv | sort | uniq -c | sort -rn > Frequency-Sponsors.csv [This contained 13,380 records]

*Method 2:*

In order to confirm that *Method 1* processed the data correctly, the following operations were performed separately, by another author, on the list of 85,528 trials:

1. filter newly downloaded list of 85,528 trials with “Drug:” OR “Biological:” in Interventions using Standard Filter in Excel. [This contained 69,168 records]

2. ssconvert file.ods file.csv [*This command converts ods to csv*]

3. sed 's/\"//g;s/|/\n/g' file.csv|sort |uniq -c |sed 's/^ *//'|sed 's/^\([^ ][^ ]*\) \(.*\)/\2\t\1/' > file.counts.tsv [*This command computes frequency*] [This yields 12,823 records]

*Sorting out two discrepancies*:

a. The number of interventions with “Drug:” OR “Biological:” by *Method 2* were 69,168 although 69,160 by *Method 1*. For some reason some rows (with multiple sponsors) were divided into two (or more) rows by *Method 2*. These were 'reassembled' correctly and then the total was 69,160 as for *Method 1*.

b. The number of unique sponsors was 13,380 by *Method 1* and 12,823 by *Method 2.* The difference occurred because of improper counting due to unidentified characters in names of organizations (due to translation from other languages). These 557 differences were identified and sorted out. The correct number of unique sponsors is 12,823.

**(C) Classifying each organization**

The **2,266** organizations in S2 Table were categorized as follows:

1. Corporates, 951 in all: (a) organizations registered as companies (that are not public-sector) and (b) other for-profit entities such as private practices.

2. Non-corporates, 1145 in all: (a) organizations that are registered as non-profits/charities (or have charity tax-status in the US), (b) consortia/networks that are funded by the government, (c) an organization that is 'public' or 'government', including public-sector companies, (d) individuals (31 cases) and (e) international organizations such as the World Bank.

3. Unclassified, 170 in all: We were unable to determine the status of several organizations and for some their location could not be determined unambiguously. These are left unclassified.

These lists are in S2 Table. To be noted is that each name is treated as a separate organization. Thus,

(i) if an organization's name has been misspelled in some instances, the different name will be considered a different organization;

(ii) if the organization has undergone a change of name, the two names will be considered two organizations;

(iii) if two organizations have merged over time, then the name of the merged entity will hold for some trials and the individual organizations for other trials.

These are all potential weaknesses of the analyses.

**(D) Determining each organization's location**

**Extensive internet searches were done to classify the 2,266 sponsors and identify the country that each is based in. Google Translate was used for websites in other languages. When this proved insufficient, emails were sent to individuals who are (or have been) associated with the organization, to friends in (or from) those countries or to the relevant embassy in New Delhi.**
